# Radionuclides in Fracking Wastewater: Managing a Toxic Blend

**DOI:** 10.1289/ehp.122-A50

**Published:** 2014-02-01

**Authors:** Valeria J. Brown

**Affiliations:** **Valerie J. Brown**, based in Oregon, has written for *EHP* since 1996. In 2009 she won a Society of Environmental Journalists’ Outstanding Explanatory Reporting award for her writing on epigenetics.

Naturally occurring radionuclides are widely distributed in the earth’s crust, so it’s no surprise that mineral and hydrocarbon extraction processes, conventional and unconventional alike, often produce some radioactive waste.^1^ Radioactive drilling waste is a form of TENORM (short for “technologically enhanced naturally occurring radioactive material”)—that is, naturally occurring radioactive material (NORM) that has been concentrated or otherwise made more available for human exposure through anthropogenic means.^2^ Both the rapidity and the extent of the U.S. natural gas drilling boom have brought heightened scrutiny to the issues of radioactive exposure and waste management.

Perhaps nowhere is the question of drilling waste more salient than in Pennsylvania, where gas extraction from the Marcellus Shale using hydraulic fracturing (fracking) made the state the fastest-growing U.S. producer between 2011 and 2012.^3^ The Marcellus is known to have high uranium content, says U.S. Geological Survey research geologist Mark Engle. He says concentrations of radium-226—a decay product of uranium—can exceed 10,000 picocuries per liter (pCi/L) in the concentrated brine trapped in the shale’s depths.

**Figure:**
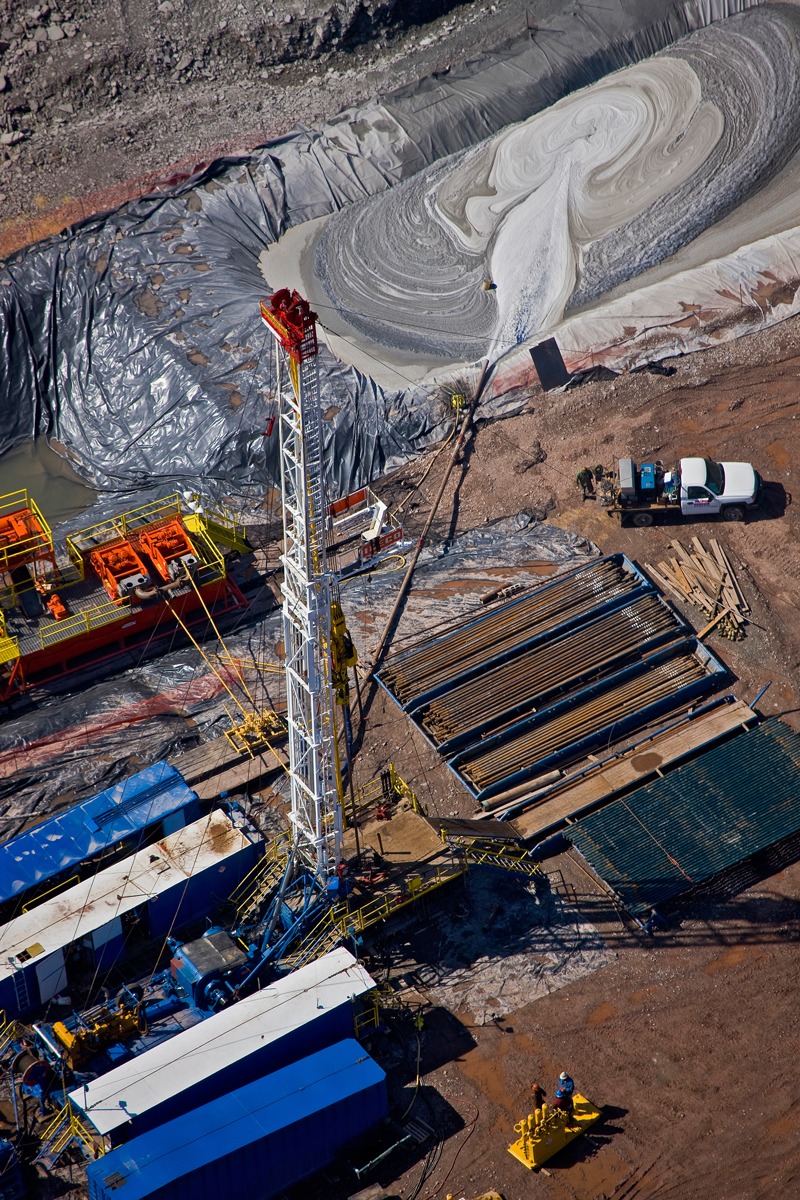
A lined impoundment receives waste at a fracking site in Dimock, Pennsylvania. © J. Henry Fair

To date the drilling industry and regulators have considered the risk posed to workers and the public by radioactive waste to be minor. In Pennsylvania, Lisa Kasianowitz, an information specialist with the state Department of Environmental Protection (PADEP), says there is currently nothing to “indicate the public or workers face any health risk from exposure to radiation from these materials.” But given the wide gaps in the data, this is cold comfort to many in the public health community.

## Waste Production and Storage

After fracking, both gas and liquids—including the injected water and any water residing in the formation (known as “flowback” and “produced water”^4^)—are pulled to the surface. Fluids trapped in the shale are remnants of ancient seawater. The salts in shale waters reached extreme concentrations over millions of years, and their chemical interactions with the surrounding rock can mobilize radionuclides.^5^^,^^6^ Several studies indicate that, generally speaking, the saltier the water, the more radioactive it is.^5^^,^^7^

Dissolved compounds often precipitate out of the water, building up as radionuclide-rich “scale” inside pipes. To remove the pipe-clogging scale, operators might inject chemicals to dissolve it.^8^ Scale also may be removed mechanically using drills, explosives, or jets of fluid,^9^ in which case it joins the solid waste stream.

Wastes are often stored temporarily in containers or in surface impoundments, also called pits and ponds. Data on how many such ponds are used in shale gas extraction are sparse, but according to Kasianowitz, there are 25 centralized impoundments in Pennsylvania. Centralized impoundments can be the size of a football field and hold at least 10 million gallons of liquid. Although at any given time the number of smaller ponds is probably much higher, she says these ephemeral lagoons are used mostly in the early phase of well development and are rapidly decommissioned.

Most impoundments are lined with plastic sheeting. Pennsylvania requires that pit liners for temporary impoundments and disposal have a minimum thickness of 30 mil and that seams be sealed to prevent leakage.^10^ Ohio’s only requirement is that pits must be “liquid tight.”^10^ However, improper liners can tear,^7^ and there have been reports of pit liners tearing and pits overflowing in Pennsylvania and elsewhere.^11^

A small 2013 study of reserve pits in the Barnett Shale region of Texas suggested another consideration in assessing pit safety. Investigators measured radium—the radionuclide generally used as a proxy to judge whether NORM waste complies with regulatory guidelines for disposal—as well as seven other radionuclides not routinely tested for. Although individual radionuclides were within existing regulatory guidelines, total beta radiation in one sample was more than 8 times the regulatory limit. “Evaluating the single radionuclide radium as regulatory exposure guidelines indicate, rather than considering all radionuclides, may indeed underestimate the potential for radiation exposure to workers, the general public, and the environment,” the authors wrote.^2^

## Surface Waters

Ultimately most wastewater is either treated and reused or sent to Class II injection wells (disposal or enhanced recovery wells). A small fraction of Pennsylvania’s fracking wastewater is still being treated and released to surface waters until treatment facilities’ permits come up for renewal under new, more stringent treatment standards, Kasianowitz says.

Concerns about NORM in the Marcellus have recently focused on surface waters in Pennsylvania. That’s because until 2011, most produced water was sent to commercial or public wastewater treatment plants before being discharged into rivers and streams, many of which also serve as drinking water supplies. In April of that year PADEP asked all Marcellus Shale fracking operations to stop sending their wastewater to treatment plants, according to Kasianowitz. Although voluntary, this request motivated most producers to begin directly reusing a major fraction of their produced water or reusing it after treatment in dedicated commercial treatment plants that are equipped to handle its contaminants.

A team of Duke University researchers led by geochemist Avner Vengosh sought to characterize the effluent being discharged from one such plant, the Josephine Brine Treatment Facility in southwestern Pennsylvania. The researchers compared radioactivity and dissolved solids in sediment both up- and downstream of the facility and found a 90% reduction in radioactivity in the effluent. The radioactive constituents didn’t just disappear; the authors noted that most had likely been transferred and accumulated to high levels in the sludge that would go to a landfill.^12^

Stream sediments at the discharge site also had high levels of radioactivity, keeping it out of the surface water downstream but posing the risk of bioaccumulation in the local food web. The outflow sediment radiation levels at the discharge site were 200 times those in upstream sediments. The study highlighted “the potential of radium accumulation in stream and pond sediments in many other sites where fracking fluids are accidentally released to the environment,” says Vengosh.

The study also demonstrated another potential impact of treated brine on water quality. Most produced water contains bromide, which can combine with naturally occurring organic matter and chlorine disinfectant to form drinking water contaminants called trihalomethanes. These compounds are associated with liver, kidney, and nervous system problems.^13^ The Duke researchers reported highly elevated concentrations of bromide over a mile downstream from the plant—a potential future burden for drinking water treatment facilities downstream.^12^

## Deep Injection

Following the 2011 policy change, Ohio’s Class II injection wells began to receive much of Pennsylvania’s end-stage wastewater. Pennsylvania’s geology does not lend itself to this method; the state has only six injection wells available for this purpose, while Ohio has 177,^10^ and Texas has 50,000.^14^

Class II injection wells place the wastewater below the rock strata containing usable groundwater. Conventional industry wisdom says this prevents migration of contaminants into shallower freshwater zones.^7^^,^^15^^,^^16^^,^^17^

But some believe this may be a flawed assumption. The reason fracking works to force gas out of the rock is also why some observers think injection wells could be unstable—the extreme pressure of injection can take nearly a year to dissipate, according to hydrologic consultant Tom Myers, who published a modeling study of fracking fluids’ underground behavior in 2012.^18^

Myers says the lingering higher-than-normal pressure could bring formation waters, along with fracking chemicals, closer to the surface far faster than would occur over natural geological time scales of thousands of years. This is particularly true if there are faults and/or abandoned wells within the fracking zone.

Another study has demonstrated the possibility that formation water can migrate into freshwater aquifers through naturally occurring pathways.^19^ Although the pathways were not, themselves, caused by gas drilling, the study authors suggest such features could make certain areas more vulnerable to contamination due to fracking.

Asked about the integrity of deep-injection wells, Vengosh says, “As far as I know nobody’s actually checking.” If such leaks were happening, he says, much would depend on how they connected to drinking water aquifers. “Unlike freshwater systems where radium would accumulate in the sediments,” he says, “if you have a condition of high salinity and reducing conditions, radium will be dissolving in the water and move with the water.”

## Beneficial Uses and Landfills

Fracking wastes may also be disposed of through “beneficial uses,” which can include applying produced water as a road de-icer or dust suppressant, using drilling cuttings in road maintenance, and spreading liquids or sludge on fields.^12^^,^^20^^,^^21^ Pennsylvania allows fracking brine to be used for road dust and ice control under a state permit.^22^ While the permit sets allowable limits for numerous constituents, radioactivity is not included.^23^

Conventional wisdom about radium’s stability in landfills rests on an assumption regarding its interaction with barite (barium sulfate), a common constituent in drilling waste. However, Charles Swann of the Mississippi Mineral Resources Institute and colleagues found evidence that radium in waste spread on fields may behave differently in soil than expected. When they mixed scale comprising radium and barite with typical Mississippi soil samples in the laboratory, radium was gradually solubilized from the barite, probably as a result of soil microbial activity. “This result,” the authors wrote, “suggests that the landspreading means of scale disposal should be reviewed.”^24^

Solids and sludges can also go to landfills. Radioactivity limits for municipal landfills are set by states, and range from 5 to 50 pCi/g.^25^ Since Pennsylvania began requiring radiation monitors at municipal landfills in 2001, says Kasianowitz, fracking sludges and solids have rarely set them off. In 2012 they accounted for only 0.5% of all monitor alarms. They “did not contain levels of radioactivity that would be acutely harmful to the public,” according to a 2012 review of Pennsylvania’s fracking practices by the nonprofit State Review of Oil and Natural Gas Environmental Regulations.^26^ Dave Allard, director of PADEP’s Bureau of Radiation Protection, points out that because all soils contain at least some radionuclides, “you’re always going to have some radium, thorium, and uranium, because these landfills are *in* soils.”

## Assessing Exposures

At the federal level, radioactive oil and gas waste is exempt from nearly all the regulatory processes the general public might expect would govern it. Neither the Atomic Energy Act of 1954 nor the Low-Level Radioactive Waste Policy Act covers NORM.^2^ The Nuclear Regulatory Commission has no authority over radioactive oil and gas waste. State laws are a patchwork. Workers are covered by some federal radiation protections, although a 1989 safety bulletin from the Occupational Safety and Health Administration noted that NORM sources of exposure “may have been overlooked by Federal and State agencies in the past.”^27^

Fracking in the Marcellus has advanced so quickly that public understanding and research on its radioactive consequences have lagged behind, and there are many questions about the extent and magnitude of the risk to human health. “We are troubled by people drinking water that [could potentially have] radium-226 in it,” says David Brown, a public health toxicologist with the Southwest Pennsylvania Environmental Health Project. “When somebody calls us and says ‘is it safe to drink our water,’ the answer is ‘I don’t know.’”

PADEP is conducting a study to determine the extent of potential exposures to radioactive fracking wastewater.^28^ The PADEP study will sample drill cuttings, produced waters, muds, wastewater recycling and treatment sludges, filter screens, extracted natural gas, scale buildup in well casings and pipelines, and waste transport equipment. PADEP will also evaluate radioactivity at well pads, wastewater treatment plants, wastewater recycling facilities, and landfills.

The EPA is studying the issue with a review of the potential impacts of hydraulic fracturing,^29^ including radioactivity, on drinking water resources. A draft of the EPA study will be released for public comment and peer review in late 2014, according to Christopher Impellitteri, chief of the Water Quality Management Branch at the agency’s National Risk Management Research Laboratory.

The EPA study includes research designed to assess the potential impacts from surface spills, well injection, and discharge of treated fracking wastewater on drinking water sources. One project will model the transport of contaminants, including radium, from treatment outflows in receiving waters. Field and laboratory experiments will characterize the fate and transport of contaminants in wastewater treatment and reuse processes. Groundwater samples are being tested for radium-226, radium-228, and gross alpha and beta radiation. The overall study does not include radon.^29^

Both radon and radium emit alpha particles, which are most dangerous when inhaled or ingested. When inhaled, radon can cause lung cancer, and there is some evidence it may cause other cancers such as leukemia.^30^ Consuming radium in drinking water can cause lymphoma, bone cancer, and leukemias.^31^ Radium also emits gamma rays, which raise cancer risk throughout the body from external exposures. Radium-226 and radium-228 have half-lives of 1,600 years and 5.75 years, respectively. Radium is known to bioaccumulate in invertebrates, mollusks, and freshwater fish,^12^ where it can substitute for calcium in bones. Radium eventually decays to radon; radon-222 has a half-life of 3.8 days.

Geochemically, radon and radium behave differently. Radon is an inert gas, so it doesn’t react with other elements and usually separates from produced water along with methane at the wellhead. Although there are few empirical data available, the natural gas industry has not been concerned about radon reaching its consumers in significant amounts, in part because of radon’s short half-life and because much of it is released to the atmosphere at the wellhead.^32^

## Beyond Assumptions

Assumptions about quality control underlie much of the debate about whether the risks of fracking outweigh the benefits. “If everything is done the way it’s supposed to be done, the impact of this radioactivity would be fairly minimal in the environment in Pennsylvania, because they’re reusing the water,” says Radisav R. Vidic, a professor of civil and environmental engineering at the University of Pittsburgh. “The only potential pathway is an accident, a spill, or a leak.” But, he adds, “That’s something that happens in every industry, so there’s nothing you can do about it.”

Indeed, Vengosh says, PADEP has reports of hundreds of cases of spills and contamination that involved fracking fluids. Furthermore, he says, “The notion that the industry can reuse all flowback and produced water is simply not possible, given the chemistry of the wastewater.”

Many of the studies to date on fracking’s environmental impacts have suffered from a lack of access to actual treatment practices, according to Engle. He attributes this to a lack of trust between the industry and scientists, and the fact that such information is often proprietary. But Swann reports a different experience working with Mississippi producers. “The small, independent producers were very willing to cooperate and gladly provided assistance, often at their expense,” he says. “Only through their assistance were we able to sample so many fields and wells.”^24^

Research published in December 2013 suggests one potential new treatment for radioactivity in fracking waste.^33^ Vengosh and colleagues combined various proportions of flowback water with acid mine drainage (AMD) to test the possibility of using the latter as an alternative source of water for fracking. AMD—acidic leachate from mining sites and other disturbed areas—is an important water pollutant in some regions. Laboratory experiments showed that mixing flowback water with AMD caused much of the NORM in the flowback to precipitate out, leaving water with radium levels close to EPA drinking water standards.

The authors suggest the radioactive precipitate could be diluted with nonradioactive waste to levels appropriate for disposal in municipal landfills. If it can be brought to industrial scale, Vengosh says, this method could provide a beneficial use for AMD while reducing the need for freshwater in fracking operations and managing the inevitable radioactive waste.

Studies such as this provide a light at the end of the wellbore. Yet the current patchy understanding of radioactive fracking waste’s fate in the environment precludes making good decisions about its management. And even if fracking the Marcellus ceased overnight, the questions and potential problems about radioactivity would linger. “Once you have a release of fracking fluid into the environment, you end up with a radioactive legacy,” says Vengosh.
