# Erratum: “Radionuclides in Fracking Wastewater: Managing a Toxic Blend”

**DOI:** 10.1289/ehp.122-A149

**Published:** 2014-06-01

The Focus article “Radionuclides in Fracking Wastewater: Managing a Toxic Blend” [Environ Health Perspect 122:A50–A55 (2014); http://dx.doi.org/10.1289/ehp.122-A50] incorrectly stated that Pennsylvania requires pit liners for temporary impoundments and disposal to have a minimum thickness of 30 mm. The correct minimum thickness is 30 mils.

*EHP* regrets the error.

